# Eosinophilic Bronchiectasis: Prevalence, Severity, and Associated Features—A Cohort Study

**DOI:** 10.3390/jcm13164932

**Published:** 2024-08-21

**Authors:** Raffaele Campisi, Santi Nolasco, Manuel Mancuso, Miriam Spinella, Fabio Vignera, Nunzio Crimi, Carlo Vancheri, Claudia Crimi

**Affiliations:** 1 Respiratory Medicine Unit, Policlinico “G. Rodolico-San Marco” University Hospital, 95123 Catania, Italy; raffaelemd@hotmail.it (R.C.); nolascos@hotmail.it (S.N.); vancheri@unict.it (C.V.); 2 Department of Clinical and Experimental Medicine, University of Catania, 95123 Catania, Italy; 95manuelmancuso@gmail.com (M.M.); miriam.spinella@gmail.com (M.S.); vignerafabio2022@gmail.com (F.V.);

**Keywords:** eosinophil, bronchiectasis, Type 2 (T2) inflammation, exacerbations

## Abstract

**Background:** Bronchiectasis (BE) has been traditionally associated with neutrophilic inflammation, but eosinophilic bronchiectasis (EB) has recently emerged. Data about prevalence, clinical features, and disease severity are lacking. This study aimed to assess the EB prevalence, compare EB with non-EB, evaluate the Type-2 (T2) high endotype in BE (T2-high EB) versus non-T2-high EB, and identify EB predictors. **Methods:** We conducted a prospective study involving 153 BE patients. The data collected included clinical, radiological, and microbiological findings. BE severity was assessed using the bronchiectasis severity index (BSI), FACED and E-FACED scores, and the bronchiectasis etiology and comorbidity index (BACI). EB was defined as a blood eosinophil count (BEC) ≥ 300 cells/μL, and T2-high EB as BEC ≥ 300 cells/μL with fractional exhaled nitric oxide (FeNO) ≥ 25 ppb. **Results:** Prevalence was 27% for EB and 20% for T2-high EB. EB patients exhibited poorer lung function and more severe radiologic features, with significantly higher severity scores [BSI, FACED, E-FACED, BACI (*p* < 0.05)], and a higher median exacerbation rate [4 (2–5) in EB vs. 2 (1–4) in non-EB, *p* = 0.0002], compared with non-EB patients. T2-high EB patients showed higher severity scores [BSI, FACED, E-FACED (*p* < 0.05)], as well as worse lung function parameters [FEV_1_%, FVC%, FEF _25–75_% (*p* < 0.05)] compared with non-T2-high EB patients. In our study, patients with EB exhibited notably worsened lung function and higher BE severity scores compared with their non-EB counterparts, with exacerbations playing a major role in these differences. We found statistically significant positive correlations between BEC and disease severity scores, such as BSI, FACED, and mMRC, as well as an inverse relationship with pulmonary function. The likelihood of EB being present was significantly higher in association with mMRC ≥ 1 (OR = 2.53; 95% CI, 1.26–5.64), exacerbations/year ≥ 1 (OR = 1.27; 95% CI, 1.0–1.63), and chronic PA colonization (OR = 3.9; 95% CI, 1.08–15.8). **Conclusions:** EB is a distinct endotype. Dyspnea, exacerbations, and PA colonization may be predictive of EB, emphasizing the importance of early detection for improved outcomes. BEC could serve as a useful biomarker of disease severity to consider when diagnosing EB.

## 1. Introduction

Bronchiectasis (BE) is a chronic respiratory disorder characterized by bronchial widening due to mucociliary dysfunction, inflammation, and infection [1]. BE exhibits variability in radiological patterns, microbial colonization, as well as in clinical and inflammatory features [2,3]. BE, previously considered an “orphan disease”, has now emerged as a debilitating condition with a growing prevalence that can affect individuals of all ages, imposing a substantial burden on healthcare systems due to its morbidity and mortality, poor quality of life, frequent exacerbations and hospitalizations [4,5]. The epidemiology of BE exhibits considerable variability, but the recent creation of national and international registries focused on this clinical condition has greatly enhanced our understanding of BE [6,7].

Neutrophils are widely recognized as the primary inflammatory cells in BE and play a pivotal role in pathogenesis, progression, and severity [8,9]. Therapies targeting neutrophils, such as inhaled antibiotics and long-term macrolides, are effective in mitigating inflammation and improving outcomes [10]. While neutrophilic inflammation is common, recent studies suggest the involvement of other inflammatory cells in disease progression [11]. Eosinophils are known for their pro-inflammatory properties and contribution to airway remodeling and damage. They have been implicated in BE pathogenesis [12] and observed in bronchial biopsies, sputum, and blood of BE patients. Eosinophils are observed in approximately 20% of cases in the absence of asthma, allergic bronchopulmonary aspergillosis (ABPA), or other eosinophilic conditions [13]. This variant of BE is referred to as eosinophilic bronchiectasis (EB) when accompanied by an elevated blood eosinophil count (BEC) [14,15,16]. Furthermore, a BE subtype characterized by high Type-2 (T2) inflammation, defined as BEC ≥ 300 eosinophils/μL, and oral fractional exhaled nitric oxide (FeNO) ≥ 25, known as T2-high EB, has been identified [17]. Recently, BEC ≥ 300 eosinophils/μL has been validated as a surrogate marker for eosinophilic inflammation in BE. This parameter is associated with disease severity, impaired lung function, increased exacerbation risk, and poor quality of life [13,18]. BEC represents a potential treatable trait with implications for precision treatments as previously explored in patients with co-existing asthma [19,20,21]. This study aimed to investigate the following: (1) Assess the prevalence, clinical characteristics, and severity of EB compared with non-EB and of T2-high EB compared with non-T2-high EB; (2) Explore correlations between BEC and the functional, clinical, and severity features of BE; (3) Evaluate the likelihood of EB based on clinical, functional, and radiological characteristics.

## 2. Materials and Methods

### 2.1. Study Population

**Figure 1:**
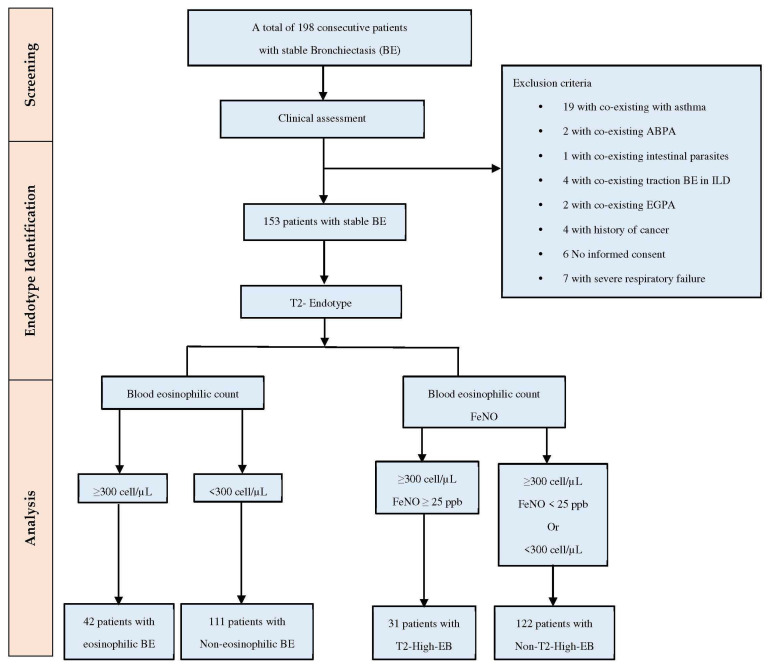
Flow diagram demonstrating patients’ selection. Abbreviations: ABPA, allergic bronchopulmonary aspergillosis; ILD, interstitial lung disease; EGPA, eosinophilic granulomatosis with polyangiitis; FeNO, fractional exhaled nitric oxide; and T2-High, T(2) high eosinophilic inflammation.

This prospective observational study included 198 stable BE patients (aged ≥ 18 years) diagnosed according to the ERS guidelines [1] and recruited between January 2022 and February 2024 at our outpatient Respiratory Unit—AOU Policlinico—San Marco, Catania. This study adhered to the Declaration of Helsinki and was approved by the “Catania 1” Ethics Committee (Approval Number 108/2018/PO). The selection process and exclusion criteria are presented in a flow diagram (Figure 1). Of the initial 198 patients, 45 were excluded due to co-existing asthma, ABPA, intestinal parasites, traction bronchiectasis in ILD, history of cancer, lack of informed consent, or respiratory failure. The remaining 153 patients selected for the study were categorized into two populations based on the T2-endotype. The population with EB was defined by BEC ≥ 300 cells/μL, whereas the non-EB group was characterized by BEC of <300 cells/μL. The T2-high EB group was classified as BEC of ≥300 cells/μL and FeNO levels of ≥25 parts per billion (ppb), while the non-T2-high EB group included patients with either BEC of ≥300 cells/μL and FeNO levels < 25 ppb or BEC of < 300 cells/μL with FeNO levels ≥ 25 ppb.

### 2.2. Data Collection

The demographics, smoking status, exacerbations and hospitalizations (up to 12 months earlier), BEC, and comorbidities [chronic obstructive pulmonary disease (COPD), chronic rhinosinusitis (CRS), sleep-disordered breathing (SDB), gastroesophageal disease (GERD), anxiety/depression, obesity, diabetes, cardiovascular disease, and history of cancer] were collected at enrollment. Laboratory data included alpha-1 antitrypsin, C-reactive protein, IgA, IgM, IgG and subclasses, vitamin D, beta-D-glucan, galactomannan, and Aspergillus antibodies by double immunodiffusion method. FeNO was measured according to the ERS/ATS guidelines [22]. Sputum samples for microbiological testing were analyzed for pathogens (bacteria, fungi, mycobacteria) during the registry visit or within the previous year. PA colonization was defined as a positive sputum culture on two or more occasions. The following respiratory symptoms were also recorded: chronic mucus hypersecretion (CMH), cough, coughing sputum, chest pain, Medical Research Council (mMRC) dyspnea score, and hemoptysis. Forced vital capacity (FVC), forced expiratory volume in the first second (FEV1), and forced expiratory flow between 25% and 75% of FVC (FEF25–75) were assessed according to the ERS/ATS guidelines [23].

### 2.3. Bronchiectasis Diagnosis and Severity Assessment

BE was diagnosed by high-resolution computed tomography (HRCT) using the following radiological criteria: inner or outer airway–artery diameter ratio of ≥1.1 (or ≥1.5 for enhanced specificity), lack of tapering, and airway visibility of up to 1 cm from the pleura [24]. A modified Reiff score [25] was used to evaluate BE considering lobe involvement (including the lingula as a separate lobe) and the extent of dilation, categorized as tubular = 1, varicose = 2, and cystic = 3, with a maximum score of 18. BE severity was assessed using the bronchiectasis severity index (BSI), FACED, and E-FACED scores. The BSI combines age, BMI, FEV1% predicted, hospitalization history (previous two years), exacerbations (past year), mMRC, radiological severity (≥3 lobes affected or presence of cystic BE), and PA or other colonization. BE was defined as mild (BSI = 0–4 points), moderate (BSI = 5–8 points), or severe (BSI ≥ 9 points) [26]. The FACED score includes FEV1% predicted (F, cutoff at 50%, maximum 2 points); age (A, cutoff at 70 years, maximum 2 points); chronic PA colonization (C, maximum 1 point); radiological involvement (E, number of affected lobes, cutoff at two lobes, maximum 1 point); and dyspnea (D, cutoff at grade II of mMRC, maximum 1 point) [27,28]. The E-FACED score also incorporates the exacerbation rate, with a threshold of ≥2 exacerbations in the previous year (an additional two points compared with FACED) [29]. The bronchiectasis etiology and comorbidity index (BACI) was used to calculate the risk of severe exacerbation requiring hospitalization, mortality, and overall disease burden with associated comorbidities. Three risk categories were established: 0 points (low risk), 1–5 points (intermediate risk), and ≥6 points (high risk) [30].

### 2.4. Statistical Analysis

Data were presented as mean ± SD or median with interquartile range (IQR) for normally and non-normally distributed variables, respectively. The normality of data distribution was assessed using the Kolmogorov–Smirnov and Shapiro–Wilk tests. Categorical variables were expressed as n (%) values. Independent *t*-tests or Mann–Whitney tests were used to compare continuous parametric and nonparametric variables. A Fisher’s exact test was run to compare categorical variables when appropriate. Linear regression analysis was used, along with Spearman’s rank correlation coefficients (ρ), to assess the relationship between BEC and the clinical, functional, and severity features of BE. Multiple logistic regression was used to calculate adjusted odds ratios (ORs) with 95% confidence intervals (CIs) and to determine the influence of clinical, microbiological, radiological, and functional variables on predicting the likelihood of EB. Statistical analyses and figures were performed using Prism version 10.1.0 (GraphPad Software Inc., San Diego, CA, USA). A *p*-value < 0.05 (two-sided) was considered statistically significant.

## 3. Results

### 3.1. Demographics and Clinical Characteristics of the Study Population

A total of 153 stable BE patients were included (Table 1). There were 85 females (55%) and 68 males (45%) with a median age of 65 (56–73.5) years and a median BMI of 24 (21–27.7) kg/m^2^. Patients had a median FEV1 of 91% (93–110), a mean FVC% of 90.5 ± 24.4, and a median FEF25–75% of 62 (31–98) of predicted. The median BEC was 170 cells/μL (100–325). BE etiology analysis showed that 51% of cases were idiopathic, 26% post-infectious, and 22% secondary to other conditions. Patients showed a mean BSI of 6 ± 3, a median FACED score of 2 (1–3), a median E-FACED score of 3 (1–4), and a median BACI score of 2 (0–5). The median number of exacerbations per year was 3 (1–4), with no hospitalizations. Comorbidities were COPD (35%), GERD (38%), arterial hypertension (44%), anxiety (20%), and chronic ischemic heart disease (32%). Clinical symptoms included dyspnea (mean mMRC 2 ± 1), CMH (16%), cough (88%), coughing sputum (73%), hemoptysis (11%), and chest pain (14%). Chronic PA infection was documented in 23% of patients. Patients were divided into two study cohorts. The first cohort was in relation to BEC: 42 [(27%), (95% CI, 12.14–24.85)] with EB and 111 [(73%), (95% CI, 39.95–55.59)] with non-EB. The second cohort was based on T2 endotype: 31 [(20%), (95% CI, 8.62–19.33)] with T2-high endotype (T2-high EB), and 122 [(80%), (95% CI, 72.68–85.34)] with non-T2-high (non-T2-high EB).

### 3.2. Relationship between BEC and BE Endotype

**Figure 2:**
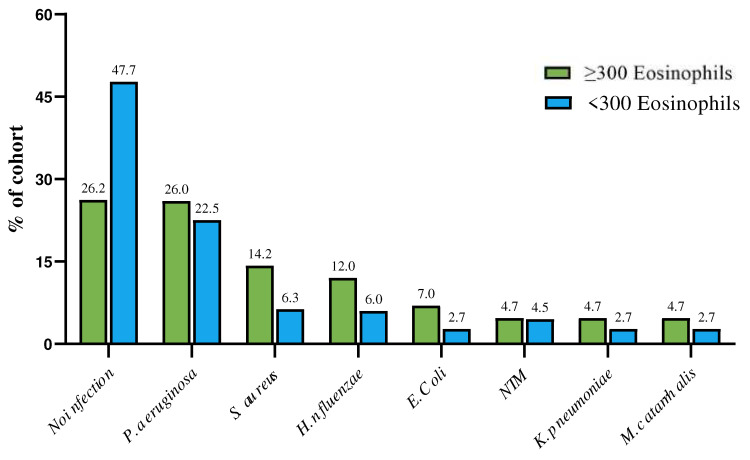
Distribution of microbiology in patients with and without EB in relation to blood eosinophilic cell count. (microbiology refers to any isolation of this pathogen in sputum during the registry visit or within the previous year).

Comparative data between the EB and non-EB populations in relation to BEC are summarized in Table 2. EB patients exhibited a significant difference in median BEC, FeNO, and all BE severity scores, such as BSI, FACED, and E-FACED, when compared with non-EB. Median modified Reiff score (*p* = 0.008), median number of lobes with BE (*p* = 0.007), secondary BE (*p* = 0.01), median exacerbation rate (*p* = 0.0002), median hospitalizations/year (*p* = 0.01), median BACI score (*p* = 0.001), COPD (52%, *p* = 0.008), and diabetes (19%, *p* = 0.02) were higher in EB compared with non-EB patients. The EB cohort had lower median FEV1% (*p* = 0.01), FVC L (*p* = 0.008), FEF 25–75% (*p* = 0.001), and FEF25–75 L (*p* = 0.001) values compared with the non-EB cohort. Significant differences in dyspnea [median mMRC 2.5 (2–3) in EB vs. 2 (1–2) in non-EB, *p* < 0.0001] and other infections [18 (42.8%) in EB vs. 28 (25%) in non-EB, *p* = 0.04] were observed. There were no differences in demographics, smoking status, other symptoms, comorbidities, PA (22.5% in non-EB vs. 26% in EB), and NTM infections. Further details on infection distribution are summarized in Figure 2. Regarding the T2-endotype (Table 3), there were no differences in demographics, etiology (idiopathic or post-infectious BE), lung function (median FEV1 L), exacerbation and hospitalization rates, comorbidities (GERD, CRS, arterial hypertension, chronic ischemic heart disease, anxiety, and depression), symptoms (CMH, cough, coughing sputum, chest pain), or microbiology (PA, NTM, and other infections). T2-high EB showed significantly higher median eosinophil count and median FeNO, as well as higher BSI, FACED, E-FACED, modified Reiff scores, and number of lobes involved, compared with non-T2-high EB patients. The T2-high EB group showed higher values for secondary BE (*p* = 0.02) and worse pulmonary function. Comorbidities such as COPD, SDB, AF, and diabetes (22%, *p* = 0.01) were prevalent in T2-high EB. A higher BACI score, worse dyspnea, and a higher proportion of patients with hemoptysis were also observed in T2-high EB.

### 3.3. Correlation between BEC and the Clinical, Radiological, and Severity Features of BE

**Figure 3:**
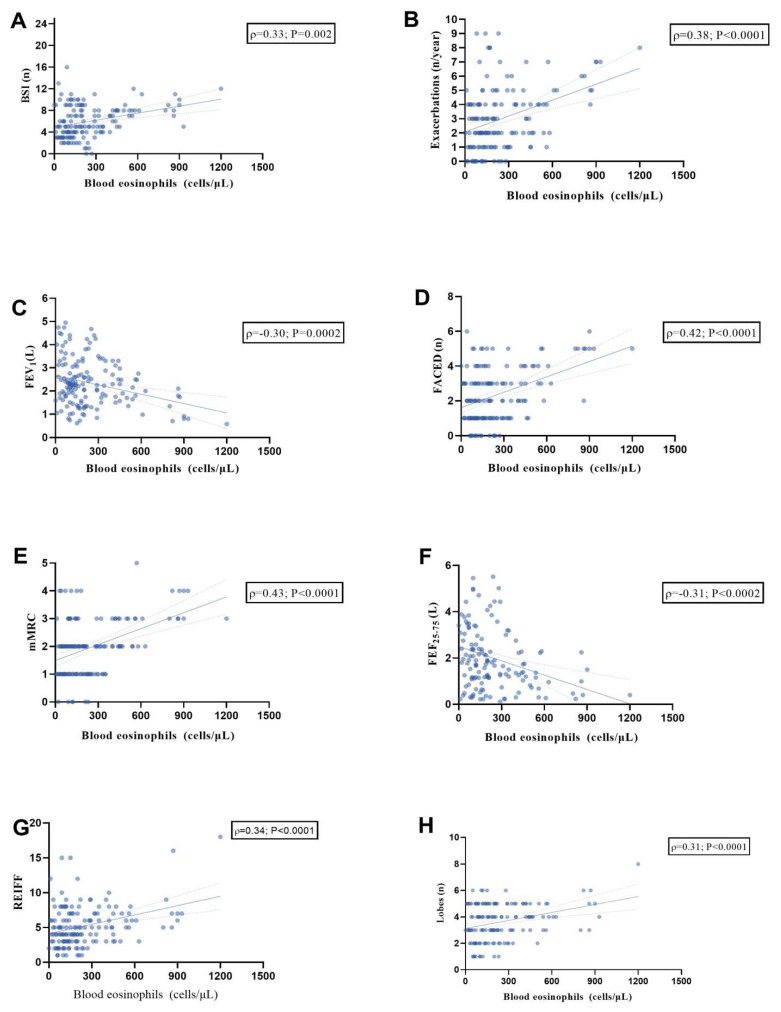
Scatter diagrams and regression lines (95% CI) showing the correlations between blood eosinophils count in the whole population with BSI (n) (**A**); exacerbations (n/year) (**B**); FEV1 (L) (**C**); FACED (n) (**D**); mMRC (**E**); FEF _25–75%_ (L) (**F**); Reiff score (**G**); Lobes (n) (**H**). All parameters are expressed as median values (IQR); r: Spearman coefficient. Abbreviations: FEV_1_, forced expiratory volume in the 1st second; BSI, bronchiectasis severity index; mMRC, modified Medical Research Council scale; and FEF_25–75_ (L), forced expiratory flow between 25% and 75% of FVC.

To investigate the potential relationship between the BEC and BE features, we analyzed BEC in 153 patients (Figure 3). A significant positive linear relationship was observed between BEC and BSI (Figure 3A; r = 0.33, *p* = 0.002), exacerbations/year (Figure 3B; r = 0.38, *p* < 0.0001), FACED score (Figure 3D; r = 0.42, *p* < 0.0001), mMRC dyspnea scale (Figure 3E; r = 0.43, *p* < 0.0001), Reiff score (Figure 3G; r = 0.34, *p* < 0.0001), and affected lobes (Figure 3H; r = 0.31, *p* < 0.0001).

These findings suggest that higher eosinophil counts may be associated with more severe symptoms and greater disease burden. Additionally, an inverse relationship was observed with FEV_1_ L (Figure 3C; r = −0.30, *p* = 0.0002) and FEF_25–75_% (Figure 3F; r = −0.31, *p* = 0.0002), suggesting a correlation between worse lung function and elevated BEC.

### 3.4. Multiple Logistic Regression Analysis to Predict the Odds Ratios of EB

**Figure 4:**
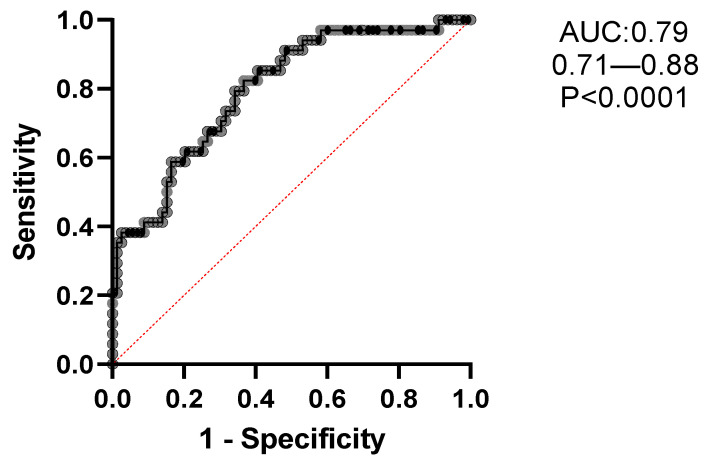
Receiver operating characteristics (ROC) of the optimal multiple regression model of variables [age, sex (F), BMI (kg/m^2^), COPD, chronic rhinosinusitis, BACI, mMRC, hemoptysis, BSI, FACED, exacerbations/year, chronic Pseudomonas infection and FEV_1_ (%)], strongly associated with comorbid eosinophilic bronchiectasis. The resulting accuracy of this model using the area under the curve (AUC) of the receiver operating characteristics (ROC) was 79% (95% CI: 71–88), with a specificity of 77% and a sensibility of 65%, *p* < 0.0001.

A multiple logistic regression analysis was conducted to evaluate the likelihood of EB, as described in Table 4. Adjusted odds ratios (with corresponding 95% CIs) were calculated for clinical, radiological, functional, and microbiological variables, including age, sex (female), BMI, COPD, presence of CRS, mMRC dyspnea scale, hemoptysis, BACI, BSI, and FACED scores, exacerbations/year, chronic PA colonization, and FEV1%. The likelihood of EB being present was significantly higher in association with mMRC ≥ 1 (OR = 2.53; 95% CI, 1.26–5.64), exacerbations/year ≥ 1 (OR = 1.27; 95% CI, 1.0–1.63), and chronic PA colonization (OR = 3.9; 95% CI, 1.08–15.8). The resulting accuracy of this model, based on the area under the curve (AUC) of the receiver operating characteristics (ROC), was 79% (95% CI, 71–88), with a specificity of 77% and a sensitivity of 65% (*p* < 0.0001); see Figure 4.

## 4. Discussion

In this prospective observational study of 153 clinically stable patients with BE, we supported the hypothesis of a distinct and well-defined eosinophilic endotype in BE. The main findings of this study are as follows: (1) Both patient groups exhibited worse lung function, BSI, FACED, E-FACED, BACI scores, and increased exacerbations/year, as well as greater radiological severity; (2) Higher BEC levels correlated with increased BE severity; (3) Variables associated with the likelihood of EB included chronic PA infection, dyspnea (mMRC ≥ 1), and recurrent exacerbations.

Taken together, these findings support the hypothesis of a distinct and well-defined eosinophilic endotype in BE, which is a distinct phenotype within the BE spectrum. Notably, 27% of patients in our cohort exhibited EB, and 20% exhibited T2-high EB, consistent with the findings reported in other studies [13,14,15,16,17]. These results emphasize the complexity of EB and underscore the importance of clinical assessment and proper phenotyping to identify high-risk patients [31].

Chronic inflammation plays a fundamental role in the pathophysiology of BE, with neutrophils traditionally identified as key inflammatory cells. However, eosinophils in the blood and sputum of BE patients are retrieved in up to one-third of cases [32,33,34], similar to COPD and asthma, where clinical responses to treatment based on T2-inflammation levels identify subgroups at increased risk of bacterial infections and pneumonia [35,36]. Eosinophils actively contribute to the pathogenesis of BE, with markers of eosinophilic inflammation observed even in patients without asthma [2,10]. Eosinophils release substances such as elastases, eosinophil cationic protein, and exosomes implicated in airway damage and BE development [37]. Additionally, eosinophil peroxidase and T2-associated cytokines, such as IL-4, IL-5, and IL-13, contribute to mucus production and eosinophils recruitment in BE airways. As a result, eosinophils significantly affect lung function, with EB patients exhibiting worse functional profiles than other BE subtypes [38].

Shoemark et al. analyzed cohorts of BE patients from five countries, excluding those with co-existing asthma, to investigate the role of eosinophils in BE. They demonstrated a correlation between blood and sputum eosinophil counts in BE, similar to that observed in COPD and asthma, suggesting BEC as a marker of eosinophilic airway inflammation. When exploring microbiome profiles, BEC ≥ 300 cells/mL was found to be generally associated with *Streptococcus pneumoniae* and PA infections, while BEC < 100 cells/mL correlated with a predominance of *Haemophilus influenzae* and *Moraxella catarrhalis*. Likewise, a post hoc analysis of 144 patients with PA infection from the PROMIS phase 2 trial showed that elevated BEC represents a risk factor for exacerbations [13]. Therefore, the predictive value of BEC for exacerbations may mainly apply to BE patients with more frequent exacerbations or PA colonization [14]. In line with our findings, Martínez-García et al. examined the link between BEC in BE patients and the frequency and severity of exacerbations, as well as their responses to inhaled corticosteroids (ICSs). They observed a U-shaped correlation between BEC levels and severity, exacerbations, lung function, microbiological profile, and ICS treatment, thus indicating a greater disease severity and a higher exacerbation rate in patients with eosinopenia (BEC < 50 cells/µL) and eosinophilia (BEC > 300 cells/µL), versus those with low or normal BEC (51–300 cells/µL). Furthermore, ICSs reduced both the frequency and severity of exacerbations, particularly in patients with high BEC [11]. Oriano et al. further analyzed the BE endotype by considering both BEC ≥ 300 cells/µL and FeNO ≥ 25 ppb and found that T2-high EB was present in 31% of patients who exhibited more severe disease or worse symptoms [17].

Clinical complexity, higher exacerbation rates, and infections emphasize the substantial burden of EB, as evidenced by prolonged hospitalizations and significantly higher healthcare costs among EB versus non-EB patients [39]. Chen et al. evaluated hospitalized patients with BE exacerbations based on BEC levels. EB patients exhibited worse lung function and greater severity, as evidenced by higher BSI and E-FACED scores, as well as higher hospitalization costs [16]. These findings are consistent with those reported by Kwok et al., who demonstrated that BEC values of 250 cells/µL during a stable state can be a significant cutoff for hospitalizations due to BE exacerbations [40]. In addition, Ren et al. confirmed that serum total IgE and BEC were associated with the radiological extent and severity of BE, thus highlighting the necessity for further research on the role of T2 inflammation in BE [41]. Martínez-García and coworkers also assessed BEC stability over time. Their cohort was unaffected by ICSs in BE patients with no other eosinophilic diseases during the first year of observation, with a significant decrease from the second year of measurement onwards. Consequently, BEC monitoring should be periodically assessed [42].

The identification of EB as a distinct endotype suggests that while the management of neutrophilic-predominant BE typically relies on airway clearance techniques, antibiotic therapy, and addressing underlying causes, EB requires personalized treatment strategies. Both ICSs and biological therapies have shown significant value in managing severe asthma and COPD. These therapies primarily target T2-high inflammation, thus indicating their potential usefulness in EB, as well. For instance, in BE patients with BEC ≥ 300 cells/μL, the use of ICSs such as fluticasone has been shown to reduce severe exacerbations and enhance quality of life [43]. Biologics targeting eosinophils, such as mepolizumab and benralizumab, improve severe eosinophilic asthma outcomes, although randomized, placebo-controlled trials in EB are lacking. Nevertheless, case series involving BE patients (without asthma but with high BEC and recurrent exacerbations) have shown promising results with biologics, including reduced BEC, improved lung function, dyspnea, and fewer exacerbations [44]. Similar outcomes were observed in severe asthma with co-existing BE [19,20] and COPD characterized by a T2-high inflammation, thus underscoring the effectiveness of tailored eosinophil-directed therapies [45,46,47]. Clearly, identifying biomarkers and conducting randomized clinical trials are particularly important within EB patient cohorts.

This study has some limitations. First, the sample size is relatively small, paving the way for potential unmeasured confounding factors, namely variables that were not captured or adequately controlled. Secondly, our study was conducted at a single site offering standardized protocols, which may imply selection bias.

Our study’s strengths include the prospective design and the exclusion of patients with other diseases often linked to eosinophilia, as well as the identification of endotypic and phenotypic characteristics of EB patients. Additionally, we identified specific clinical and microbiological features as predictors of EB. This approach offers insights into prevention strategies in high-risk patients and future-tailored therapeutic approaches.

## 5. Conclusions

In conclusion, the multifaceted landscape of EB suggests an era where clinical insights and therapies converge seamlessly. While neutrophilic inflammation prevails in BE, eosinophils may help identify patients with distinct characteristics and potential responsiveness to targeted treatments. Biologics such as mepolizumab, benralizumab, and others signify a shift in treatment approaches, addressing challenges in BE and supporting further research to elucidate eosinophilic inflammation in BE and explore therapy effectiveness.

## Figures and Tables

**Table 1:** Baseline demographic and clinical features of patients.

Clinical Features	Patients (*n* = 153)
**Demographics**	
Sex (female), n (%)	85 (55)
Age, median (IQR) years	65 (56–73.5)
Former/Active smokers, n (%)	85 (55)
BMI [kg/m^2^], median (IQR)	24 (21–27.7)
**Biomarkers**	
Eosinophils cells/µL, median (IQR)	170 (100–325)
FeNO, ppd, median (IQR)	22 (11–31)
**Disease severity**	
BSI, mean (SD)	6 ± 3
FACED, median (IQR)	2 (1–3)
E-FACED, median (IQR)	3 (1–4)
**Radiology**	
Reiff score, median (IQR)	5 (3–7)
Number of Lobes with BE, mean (SD)	3.6 (1.3)
**Etiology**	
Idiopathic, n (%)	79 (51)
Post-infective, n (%)	40 (26)
Secondary, n (%)	34 (22)
● COPD	22 (64)
● Immunodeficiency	3 (9)
● Ciliary dysfunction	3 (9)
● Congenital malformation	1 (3)
● Aspiration/oesophageal reflux	5 (15)
**Pulmonary Function**	
FEV_1_, %, median (IQR) predict.	91 (93–110)
FEV_1_, L, median (IQR)	2.16 (1.5–2.7)
FVC, %, mean (SD) predict.	90.5 ± 24.4
FVC, L, median (IQR)	3.2 (2.4–4.6)
FEF_25–75_, %, median (IQR) predict.	62 (31–98)
FEF_25–75_, L, median (IQR)	1.5 (0.8–2.5)
**Comorbidities**	
COPD, n (%)	54 (35)
GERD, n (%)	59 (38)
Sleep Disorder Breathing, n (%)	15 (9)
Chronic Rhinosinusitis, n (%)	26 (17)
Arterial Hypertension, n (%)	68 (44)
Chronic Ischemic Heart Disease, n (%)	49 (32)
Atrial Fibrillation, n (%)	5 (3)
Diabetes, n (%)	14 (9)
Anxiety, n (%)	31 (20)
Depression, n (%)	13 (8)
**Clinical Status**	
Exacerbations/year, median (IQR)	3 (1–4)
Hospitalizations/year, media (IQR)	0 (0–0)
BACI, median (IQR)	2 (0–5)
**Symptoms**	
Dyspnea (mMRC), mean (SD)	2 ± 1
Chronic Mucus Hypersecretion, n (%)	25 (16)
Hemoptysis, n (%)	17 (11)
Cough, n (%)	135 (88)
Coughing sputum, n (%)	113 (73)
Chest pain, n (%)	22 (14)
**Microbiology**	
Pseudomonas aeruginosa, n (%)	36 (23)
NTM infections, n (%)	7 (4.5)
Other infections, n (%)	46 (30)

Data are shown as n (%), means ± SD, or median (interquartile range). Abbreviations: BMI, Body Mass Index; FeNO, fractional exhaled nitric oxide; BSI, bronchiectasis severity index; BE, bronchiectasis; COPD, chronic obstructive pulmonary disease; GERD, gastro-esophageal reflux disease; FEV_1_, forced expiratory volume in the 1st second; FVC, forced vital capacity; FEF_25–75_, forced expiratory flow between 25% and 75% of FVC; BACI, bronchiectasis etiology and comorbidity index; mMRC, modified Medical Research Council scale; and NTM, non-tuberculous mycobacteria.

**Table 2:** Baseline demographic and clinical characteristics of patients stratified by blood eosinophils count.

Total Population (n = 153)	Blood Eosinophils Count		*p* Value
	<300 cells/µL (n = 111 non-EB)	≥300 cells/µL (n = 42 EB)	
**Demographics**			
Sex (female), n (%)	61 (54.9)	24 (57.1)	0.85
Age, median (IQR) years	65 (54–73.0)	67 (59–76)	0.16
Former/Active smokers, n (%)	58 (52.2)	27 (65.1)	0.79
BMI [kg/m^2^], median (IQR)	24 (21–27.7)	25.1 (21–27.8)	0.69
**Biomarkers**			
Eosinophils cells/µL, median (IQR)	120 (70–190)	455 (300–615)	**<0.0001**
FeNO, ppd, median (IQR)	16.5 (8.75–23.25)	32 (25–43)	**<0.0001**
**Disease severity**			
BSI, median (IQR)	5 (3–9)	7.5 (5–8)	**0.001**
FACED, median (IQR)	2 (1–3)	3 (2–4)	**0.002**
E-FACED, median (IQR)	2 (1–4)	4 (1–6)	**0.02**
**Radiology**			
Reiff score, median (IQR)	4 (3–7)	6 (5–7)	**0.008**
Number of Lobes with BE, median (IQR)	3 (2–5)	4 (3–5)	**0.007**
**Etiology**			
Idiopathic, n (%)	62 (56)	17 (40.4)	0.10
Post-infective, n (%)	30 (27)	10 (24)	0.54
Secondary, n (%)	19 (17)	15 (35)	**0.01**
**Pulmonary Function**			
FEV_1_, %, median (IQR) predict.	96 (65–114)	78 (50–99.7)	**0.01**
FEV_1_, L, median (IQR)	2.23 (1.50–2.92)	2 (1.64–2.75)	0.51
FVC, %, median (IQR) predict.	95 (75.5–107)	80.5 (62.5–105.5)	0.06
FVC, L, median (IQR)	3.44 (2.5–5.3)	2.70 (2.2–3.7)	**0.008**
FEF_25–75_, %, median (IQR) predict.	69 (38–106)	41 (15.5–78)	**0.001**
FEF_25–75_, L, median (IQR)	1.87 (1.02–3.57)	1.26 (0.59–1.75)	**0.001**
**Comorbidities**			
COPD, n (%)	32 (28.8)	22 (52.3)	**0.008**
GERD, n (%)	39 (35.1)	20 (47.6)	0.19
Sleep Disorder Breathing, n (%)	8 (67.2)	7 (16.7)	0.12
Chronic Rhinosinusitis, n (%)	22 (19.8)	4 (9.5)	0.15
Arterial Hypertension, n (%)	46 (41.4)	22 (52.3)	0.27
Chronic Ischemic Heart Disease, n (%)	33 (29.7)	16 (38)	0.33
Atrial Fibrillation, n (%)	2 (1.8)	3 (7.3)	0.12
Diabetes, n (%)	6 (5.41)	8 (19)	**0.02**
Anxiety, n (%)	23 (20.7)	8 (19)	>0.99
Depression, n (%)	10 (9)	3 (7)	>0.99
**Clinical Status**			
Exacerbations/year, median (IQR)	2 (1–4)	4 (2–5)	**0.0002**
Hospitalizations/year, media (IQR)	0 (0–0)	0 (0–1)	**0.01**
BACI, median (IQR)	0 (0–5)	5 (0–7)	**0.001**
**Symptoms**			
Dyspnea (mMRC), median (IQR)	2 (1–2)	2.5 (2–3)	**<0.0001**
Chronic Mucus Hypersecretion, n (%)	12 (11.8)	13 (30.2)	0.07
Hemoptysis, n (%)	9 (8.1)	8 (19)	0.08
Cough, n (%)	99 (89.1)	36 (85.7)	0.57
Coughing sputum, n (%)	81 (71.6)	32 (76.1)	0.83
Chest pain, n (%)	12 (10.8)	10 (23.8)	0.06
**Microbiology**			
Pseudomonas aeruginosa, n (%)	25 (22.5)	11 (26.1)	0.67
NTM infections, n (%)	5 (4)	2 (6.5)	0.63
Other infections, n (%)	28 (25)	18 (42.8)	**0.04**

Data are shown as means ± SD or median (interquartile range). An unpaired Student’s *t*-test or Mann–Whitney test was used to compare continuous parametric and nonparametric variables. Fisher’s exacts test was used to compare categorical variables. Statistically significant *p*-values > 0.05 are highlighted in bold. Abbreviations: BMI, body mass index; FeNO, fractional exhaled nitric oxide; BSI, bronchiectasis severity index; BE, bronchiectasis; COPD, chronic obstructive pulmonary disease; GERD, gastro-esophageal reflux disease; FEV_1_, forced expiratory volume in the 1st second; FVC, forced vital capacity; FEF_25–75_, forced expiratory flow between 25% and 75% of FVC; BACI, bronchiectasis etiology and comorbidity index; mMRC, modified Medical Research Council scale; and NTM, non-tuberculous mycobacteria.

**Table 3:** Clinical characteristics of patients stratified in relation to T2 endotype.

Total Population (n = 153)	Non-T2-High EB (n = 122)	T2-High EB (n = 31)	*p* Value
**Demographics**			
Sex (female), n (%)	68 (55)	19 (61)	0.68
Age, median (IQR) years	65 (55–73.0)	67 (59–77)	0.24
Former/Active smokers, n (%)	66 (54)	20 (64.5)	0.31
BMI [kg/m^2^], median (IQR)	24 (21.5–27.6)	25 (21–28)	0.77
**Biomarkers**			
Eosinophils cells/µL, median (IQR)	140 (80–210)	450 (350–610)	**<0.0001**
FeNO, ppd, median (IQR)	18 (9.5–25)	39 (31–45)	**<0.0001**
**Disease severity**			
BSI, median (IQR)	5 (3–9)	8 (5–8)	**0.01**
FACED, median (IQR)	2 (1–3)	3 (2–4)	**0.004**
E-FACED, median (IQR)	2 (1–4)	4 (1–5)	**0.03**
**Radiology**			
Reiff score, median (IQR)	4 (3–7)	4 (3–5)	**0.03**
Number of Lobes with BE, median (IQR)	3 (2–5)	4 (3–5)	**0.04**
**Etiology**			
Idiopathic, n (%)	64 (52)	15 (48)	0.69
Post-infective, n (%)	35 (28.5)	5 (16)	0.17
Secondary, n (%)	22 (18)	12 (39)	**0.02**
**Pulmonary Function**			
FEV_1_, %, median (IQR) predict.	95 (65–112)	77 (50–100)	**0.03**
FEV_1_, L, median (IQR)	2.05 (1.68–2.78)	2.2 (1.50–2.80)	0.59
FVC, %, mean (SD) predict.	92 ± 24	81.6 ± 23	**0.02**
FVC, L, median (IQR)	3.2 (2.4–5.2)	2.7 (2.1–3.7)	**0.009**
FEF_25–75_, %, median (IQR) predict.	66 (35–101)	44 (15–78)	**0.02**
FEF_25–75_, L, median (IQR)	1.78 (0.91–3.39)	1.30 (0.68–1.75)	**0.01**
**Comorbidities**			
COPD, n (%)	37 (30)	17 (54)	**0.01**
GERD, n (%)	42 (34)	17 (53)	0.06
Sleep Disorder Breathing, n (%)	8 (6.5)	7 (21.8)	**0.01**
Chronic Rhinosinusitis, n (%)	23 (18.8)	2 (6)	0.10
Arterial Hypertension, n (%)	55 (45)	13 (40)	0.69
Chronic Ischemic Heart Disease, n (%)	39 (32)	10 (31)	>0.99
Atrial Fibrillation, n (%)	3 (2.4)	4 (12)	**0.03**
Diabetes, n (%)	8 (6.5)	7 (22)	**0.01**
Anxiety, n (%)	25 (20.4)	6 (18.7)	>0.99
Depression, n (%)	10 (8.2)	3 (9)	0.73
**Clinical Status**			
Exacerbations/year, median (IQR)	2 (1–4)	4 (2–5)	0.06
Hospitalizations/year, media (IQR)	0 (0–0)	0 (0–0.25)	0.60
BACI, median (IQR)	2 (0–5)	5 (0–5)	**0.04**
**Symptoms**			
Dyspnea (mMRC), median (IQR)	2 (1–2)	2 (2–3)	**0.008**
Chronic Mucus Hypersecretion, n (%)	12 (10)	6 (19)	0.21
Hemoptysis, n (%)	9 (7.3)	8 (25)	**0.01**
Cough, n (%)	108 (88.5)	27 (87)	0.76
Coughing sputum, n (%)	89 (73)	24 (77)	0.81
Chest pain, n (%)	16 (13)	6 (19)	0.39
**Microbiology**			
Pseudomonas aeruginosa, n (%)	27 (22)	9 (29)	0.47
NTM infections, n (%)	6 (5.51)	1 (2.38)	0.67
Other infections, n (%)	33 (27)	13 (42)	0.07

Data are shown as means ± SD or median (interquartile range). An unpaired Student’s *t*-test or Mann–Whitney test was used to compare continuous parametric and nonparametric variables. Fisher’s exacts test was used to compare categorical variables. Statistically significant *p*-values > 0.05 are highlighted in bold. Abbreviations: BMI, body mass index; FeNO, fractional exhaled nitric oxide; BSI, bronchiectasis severity index; BE, bronchiectasis; COPD, chronic obstructive pulmonary disease; GERD, gastro-esophageal reflux disease; FEV_1_, forced expiratory volume in the 1st second; FVC, forced vital capacity; FEF_25–75_, forced expiratory flow between 25% and 75% of FVC; BACI, bronchiectasis etiology and comorbidity index; mMRC, modified Medical Research Council scale; and NTM, non-tuberculous mycobacteria.

**Table 4:** Adjusted odds ratios (ORs) (95% CI) for age, sex (F), BMI (kg/m^2^), COPD, chronic rhinosinusitis, BACI, mMRC scale, hemoptysis, BSI, FACED, exacerbations/year, chronic Pseudomonas infection and FEV_1_ (%) in association with the presence of eosinophils ≥ 300 cells/μL, using the multiple logistic regression model.

Variable	b	S.E.	Adj. Odd Ratio	95% CI	*p* Value
Age (Y)	−0.004	2.202	0.9952	0.9547 to 1.038	0.81
Sex (F)	−0.383	0.021	0.6816	0.2361 to 1.927	0.47
BMI (kg/m^2^)	−0.016	0.531	0.9833	0.8840 to 1.090	0.74
COPD	0.094	0.052	1.099	0.2002 to 5.651	0.91
Chronic rhinosinusitis	−0.177	0.842	0.8372	0.1703 to 3.453	0.81
BACI	0.302	0.752	1.354	1.002 to 1.894	0.06
**Dyspnea–mMRC**	**0.930**	**0.161**	**2.535**	**1.269 to 5.645**	**0.01**
Hemoptysis	0.385	0.376	1.470	0.3202 to 6.321	0.60
BSI (n)	−0.176	0.750	0.8382	0.6261 to 1.096	0.21
FACED (n)	−0.128	0.141	0.8798	0.5607 to 1.338	0.56
**Exacerbations (n/year)**	**0.244**	**0.219**	**1.276**	**1.009 to 1.639**	**0.04**
**Chronic Pseudomonas infection**	**1.367**	**0.122**	**3.925**	**1.058 to 15.81**	**0.04**
FEV_1_ % pred.	0.002	0.681	1.002	0.9799 to 1.026	0.83

Dependent variable: Eosinophils < 300 cells/μL vs. Eosinophils ≥ 300 cells/μL. Cut point used: FEV_1_%: < 80%; exacerbations/year ≥ 1; mMRC ≥ 1. Abbreviations: S.E: standard error; CI, confidence interval; BMI, body mass index; COPD, chronic obstructive pulmonary disease; BACI, bronchiectasis etiology and comorbidity index; mMRC, modified Medical Research Council scale; BSI, bronchiectasis severity index; and FEV_1_, forced expiratory volume in the 1st second.

## Data Availability

The data presented in this study are available on reasonable request from the corresponding author.

## References

[1] Polverino E., Goeminne P.C., McDonnell M.J., Aliberti S., Marshall S.E., Loebinger M.R., Murris M., Cantón R., Torres A., Dimakou K. (2017). European Respiratory Society guidelines for the management of adult bronchiectasis. Eur. Respir. J..

[2] Tsikrika S., Dimakou K., Papaioannou A.I., Hillas G., Thanos L., Kostikas K., Loukides S., Papiris S., Koulouris N., Bakakos P. (2017). The role of non-invasive modalities for assessing inflammation in patients with non-cystic fibrosis bronchiectasis. Cytokine.

[3] Flume P.A., Chalmers J.D., Olivier K.N. (2018). Advances in bronchiectasis: Endotyping, genetics, microbiome, and disease heterogeneity. Lancet.

[4] Chalmers J.D., Chang A.B., Chotirmall S.H., Dhar R., McShane P.J. (2018). Bronchiectasis. Nat. Rev. Dis. Prim..

[5] Long M.B., Chotirmall S.H., Shteinberg M., Chalmers J.D. (2024). Rethinking bronchiectasis as an inflammatory disease. Lancet Respir. Med..

[6] Aliberti S., Sotgiu G., Lapi F., Gramegna A., Cricelli C., Blasi F. (2020). Prevalence and incidence of bronchiectasis in Italy. BMC Pulm. Med..

[7] Chandrasekaran R., Mac Aogáin M., Chalmers J.D., Elborn S.J., Chotirmall S.H. (2018). Geographic variation in the aetiology, epidemiology and microbiology of bronchiectasis. BMC Pulm. Med..

[8] Martínez-García M.Á., Oscullo G., Gomez-Olivas J.D. (2023). Peripheral cellular biomarkers in bronchiectasis. Respir. Med. Res..

[9] O’Donnell A.E. (2022). Bronchiectasis—A Clinical Review. N. Engl. J. Med..

[10] Martinez-Garcia M.A. (2023). Eosinophils in bronchiectasis: Searching for a new endotype. Int. J. Tuberc. Lung Dis..

[11] Keir H.R., Chalmers J.D. (2022). Bronchiectasis enters the inflammation era. Respirology.

[12] Pollock J., Goeminne P.C. (2023). Eosinophils in Bronchiectasis. Chest.

[13] Shoemark A., Shteinberg M., De Soyza A., Haworth C.S., Richardson H., Gao Y., Perea L., Dicker A.J., Goeminne P.C., Cant E. (2022). Characterization of Eosinophilic Bronchiectasis: A European Multicohort Study. Am. J. Respir. Crit. Care Med..

[14] Guan W., Oscullo G., He M., Xu D., Gómez-Olivas J.D., Martinez-Garcia M.A. (2022). Significance and Potential Role of Eosinophils in Non-Cystic Fibrosis Bronchiectasis. J. Allergy Clin. Immunol. Pract..

[15] Martínez-García M.Á., Méndez R., Olveira C., Girón R., García-Clemente M., Máiz L., Sibila O., Golpe R., Rodríguez-Hermosa J.L., Barreiro E. (2023). The U-Shaped Relationship Between Eosinophil Count and Bronchiectasis Severity. Chest.

[16] Chen W., Ran S., Li C., Li Z., Wei N., Li J., Li N. (2024). Elevated Eosinophil Counts in Acute Exacerbations of Bronchiectasis: Unveiling a Distinct Clinical Phenotype. Lung.

[17] Oriano M., Gramegna A., Amati F., D’Adda A., Gaffuri M., Contoli M., Bindo F., Simonetta E., Di Francesco C., Santambrogio M. (2021). T2-High Endotype and Response to Biological Treatments in Patients with Bronchiectasis. Biomedicines.

[18] Wang X., Villa C., Dobarganes Y., Olveira C., Girón R., García-Clemente M., Máiz L., Sibila O., Golpe R., Menéndez R. (2021). Phenotypic Clustering in Non-Cystic Fibrosis Bronchiectasis Patients: The Role of Eosinophils in Disease Severity. Int. J. Environ. Res. Public Health.

[19] Crimi C., Campisi R., Nolasco S., Cacopardo G., Intravaia R., Porto M., Impellizzeri P., Pelaia C., Crimi N. (2021). Mepolizumab effectiveness in patients with severe eosinophilic asthma and co-presence of bronchiectasis: A real-world retrospective pilot study. Respir. Med..

[20] Campisi R., Nolasco S., Pelaia C., Impellizzeri P., D’Amato M., Portacci A., Ricciardi L., Scioscia G., Crimi N., Scichilone N. (2023). Benralizumab Effectiveness in Severe Eosinophilic Asthma with Co-Presence of Bronchiectasis: A Real-World Multicentre Observational Study. J. Clin. Med..

[21] Ferri S., Crimi C., Campisi R., Cacopardo G., Paoletti G., Puggioni F., Crimi N., Heffler E. (2022). Impact of asthma on bronchiectasis severity and risk of exacerbations. J. Asthma.

[22] Exhaled N.O. (2005). ATS/ERS Recommendations for Standardized Procedures for the Online and Offline Measurement of Exhaled Lower Respiratory Nitric Oxide and Nasal Nitric Oxide, 2005. Am. J. Respir. Crit. Care Med..

[23] Miller M.R., Hankinson J., Brusasco V., Burgos F., Casaburi R., Coates A., Crapo R., Enright P., van der Grinten C.P.M., Gustafsson P. (2005). Standardisation of spirometry. Eur. Respir. J..

[24] Aliberti S., Goeminne P.C., O’Donnell A.E., Aksamit T.R., Al-Jahdali H., Barker A.F., Blasi F., Boersma W.G., Crichton M.L., De Soyza A. (2022). Criteria and definitions for the radiological and clinical diagnosis of bronchiectasis in adults for use in clinical trials: International consensus recommendations. Lancet Respir. Med..

[25] Reiff D.B., Wells A.U., Carr D.H., Cole P.J., Hansell D.M. (1995). CT findings in bronchiectasis: Limited value in distinguishing between idiopathic and specific types. Am. J. Roentgenol..

[26] Chalmers J.D., Goeminne P., Aliberti S., McDonnell M.J., Lonni S., Davidson J., Poppelwell L., Salih W., Pesci A., Dupont L.J. (2014). The Bronchiectasis Severity Index. An International Derivation and Validation Study. Am. J. Respir. Crit. Care Med..

[27] Martinez-Garcia M.A., de Gracia J., Vendrell Relat M., Giron R.-M., Maiz Carro L., de la Rosa Carrillo D., Olveira C. (2014). Multidimensional approach to non-cystic fibrosis bronchiectasis: The FACED score. Eur. Respir. J..

[28] Costa J.C., Machado J.N., Ferreira C., Gama J., Rodrigues C. (2018). The Bronchiectasis Severity Index and FACED score for assessment of the severity of bronchiectasis. Pulmonology.

[29] Martinez-Garcia M.A., Athanazio R.A., Girón R.M., Máiz-Carro L., de la Rosa D., Olveira C., de Gracia J., Vendrell M., Prados-Sánchez C., Gramblicka G. (2017). Predicting high risk of exacerbations in bronchiectasis: The E-FACED score. Int. J. Chron. Obstruct. Pulmon. Dis..

[30] McDonnell M.J., Aliberti S., Goeminne P.C., Restrepo M.I., Finch S., Pesci A., Dupont L.J., Fardon T.C., Wilson R., Loebinger M.R. (2016). Comorbidities and the risk of mortality in patients with bronchiectasis: An international multicentre cohort study. Lancet Respir. Med..

[31] Chessari C., Simonetta E., Amati F., Nigro M., Stainer A., Sotgiu G., Puci M., Gramegna A., Blasi F., Morlacchi L.C. (2024). Diagnostic delay in bronchiectasis: An Italian perspective. ERJ Open Res..

[32] Keir H.R., Chalmers J.D. (2021). Pathophysiology of Bronchiectasis. Semin. Respir. Crit. Care Med..

[33] Ferri S., Crimi C., Heffler E., Campisi R., Noto A., Crimi N. (2019). Vitamin D and disease severity in bronchiectasis. Respir. Med..

[34] Oscullo G., Gomez-Olivas J.D., Martínez-García M.Á. (2023). Eosinophilic bronchiectasis and therapeutic opportunities. Ann. Allergy, Asthma Immunol..

[35] Higham A., Beech A., Wolosianka S., Jackson N., Long G., Kolsum U., Southworth T., Pham T., Sridhar S., McCrae C. (2021). Type 2 inflammation in eosinophilic chronic obstructive pulmonary disease. Allergy.

[36] Martinez-Garcia M.A., Faner R., Oscullo G., de la Rosa D., Soler-Cataluña J.-J., Ballester M., Agusti A. (2020). Inhaled Steroids, Circulating Eosinophils, Chronic Airway Infection, and Pneumonia Risk in Chronic Obstructive Pulmonary Disease. A Network Analysis. Am. J. Respir. Crit. Care Med..

[37] Kuwabara Y., Kobayashi T., D’Alessandro-Gabazza C.N., Toda M., Yasuma T., Nishihama K., Takeshita A., Fujimoto H., Nagao M., Fujisawa T. (2018). Role of Matrix Metalloproteinase-2 in Eosinophil-Mediated Airway Remodeling. Front. Immunol..

[38] Frøssing L., Von Bülow A., Porsbjerg C. (2023). Bronchiectasis in severe asthma is associated with eosinophilic airway inflammation and activation. J. Allergy Clin. Immunol. Glob..

[39] Lei C., Zeng Z., Chen F., Guo Y., Liu Y. (2024). Eosinophilic bronchiectasis increases length and cost of hospitalization: A retrospective analysis in a hospital of southern China from 2012 to 2020. BMC Pulm. Med..

[40] Kwok W.C., Ho J.C.M., Ma T.F., Lam D.C.L., Chan J.W.M., Ip M., Tam T.C.C. (2023). Risk of hospitalised bronchiectasis exacerbation based on blood eosinophil counts. Int. J. Tuberc. Lung Dis..

[41] Ren J., Chen A., Wang J., Chang C., Wang J., Sun L., Sun Y. (2023). Association of blood total immunoglobulin E and eosinophils with radiological features of bronchiectasis. BMC Pulm. Med..

[42] Martínez-García M.A., Olveira C., Girón R., García-Clemente M., Máiz L., Sibila O., Golpe R., Rodríguez-Hermosa J.L., Barreiro E., Méndez R. (2024). Reliability of blood eosinophil count in steady-state bronchiectasis. Pulmonology.

[43] Aliberti S., Sotgiu G., Blasi F., Saderi L., Posadas T., Martinez Garcia M.A. (2020). Blood eosinophils predict inhaled fluticasone response in bronchiectasis. Eur. Respir. J..

[44] Rademacher J., Konwert S., Fuge J., Dettmer S., Welte T., Ringshausen F.C. (2020). Anti-IL5 and anti-IL5Rα therapy for clinically significant bronchiectasis with eosinophilic endotype: A case series. Eur. Respir. J..

[45] Bhatt S.P., Rabe K.F., Hanania N.A., Vogelmeier C.F., Cole J., Bafadhel M., Christenson S.A., Papi A., Singh D., Laws E. (2023). Dupilumab for COPD with Type 2 Inflammation Indicated by Eosinophil Counts. N. Engl. J. Med..

[46] Bendien S.A., Kroes J.A., van Hal L.H.G., Braunstahl G.-J., Broeders M.E.A.C., Oud K.T.M., Patberg K.W., Smeenk F.W.J.M., van Veen I.H.P.A.A., Weersink E.J.M. (2023). Real-World Effectiveness of IL-5/5Ra Targeted Biologics in Severe Eosinophilic Asthma With Comorbid Bronchiectasis. J. Allergy Clin. Immunol. Pract..

[47] Crimi C., Ferri S., Crimi N. (2019). Bronchiectasis and asthma: A dangerous liaison?. Curr. Opin. Allergy Clin. Immunol..

